# Synthesis and Interfacial Activity of Novel Heterogemini Sulfobetaines in Aqueous Solution

**DOI:** 10.1007/s11743-014-1663-5

**Published:** 2014-12-31

**Authors:** Dobrawa Kwaśniewska, Katarzyna Staszak, Daria Wieczorek, Ryszard Zieliński

**Affiliations:** 1 Department of Technology and Instrumental Analysis, Faculty of Commodity Science, Poznań University of Economics, al. Niepodległości 10, 61-875 Poznan, Poland; 2 Institute of Technology and Chemical Engineering, Poznan University of Technology, pl. Skłodowskiej-Curie 2, 60-965 Poznan, Poland

**Keywords:** Sulfobetaine, Zwitterions, Surface tension

## Abstract

Three new heterogemini sulfobetaines and their chloride salts were synthesised. The interfacial activities of the obtained chlorides in aqueous solution were studied by equilibrium and dynamic surface tension measurements. The critical micelle concentration, surface excess concentration, minimum area per surfactant molecule and standard Gibbs energy of adsorption as well as micelle lifetime and diffusion coefficient were determined. The adsorption properties and micelle lifetime of these compounds significantly depend on the length of alkyl chain. The critical micelle concentration decreases with increasing chain length of the compounds considered. The values of the diffusion coefficient of *N*-alkyl-*N*-methyl-*N*-(3-sulfopropyl)-6-(*N*-alkyl-*N*-methylamino)hexylammonium chloride tend to decrease as the concentration is increased.

## Introduction

Zwitterionic surfactants are compounds which have two ionic centres of different charge in one molecule [1]. Most often the cationic part is a quaternary ammonium group. The anionic group is typically a carboxylic acid, sulfonic acid, sulfuric acid ester and phosphoric acid ester [2]. Zwitterionic surfactants exhibit pH-dependent behaviour [3]. Furthermore, many zwitterionic surfactants are mild to the skin and eyes and exhibit low toxicity. They also demonstrate high foam stability and resistance to hard water [4]. Moreover, surfactants of this type shows good biodegradability and they also alleviate skin and eye irritation better than anionic and cationic surfactants. Thanks to all these properties, these compounds, often combined with anionic or cationic surfactants, are attractive components of domestic detergents, shampoos and other personal care products [1]. Their biological activity makes them interesting for further sophisticated applications. Recently, the possibility of using zwitterionic surfactants in materials showing good compatibility with blood [5] or materials with non-fouling interfacial properties has been studied [6].

Gemini surfactants are well known and have been intensely studied since the 1990s because of their specific molecular structure and unique properties. A characteristic feature of these compounds is that they contain in one molecule two hydrophilic headgroups and hydrophobic tails [7]; sometimes they have three heads and two tails [8]. Gemini surfactants have excellent surface-active properties that are better than their monomeric counterparts [9].

A relatively new class of surfactants, which combine in one molecule the structural features of dimeric and amphoteric surfactants, are zwitterionic gemini ones. So far not many examples of these compounds have been described in the literature. The main reason for this is their difficult synthesis [10]. Xie and Feng have described the synthesis of homogemini zwitterionic surfactants containing carbobetaine groups in their structure. These compounds show lower critical micelle concentration (CMC) than the corresponding monomeric surfactants [10]. Also Yoshimura et al. [2] have focused on homogemini surfactants with two identical zwitterionic headgroups like sulfobetaines. These compounds have good surface properties like lower CMC and better ability to lower the surface tension of water in comparison with the corresponding monomeric surfactants. Generally, gemini surfactants that are symmetrical in structure are called homogemini, whereas the asymmetrical dimeric surfactants are called heterogemini. The synthesis of the latter was recently proposed by Nyuta et al. [7]. Development of new homo- and heterogemini compounds is a new trend in surfactants. This paper concerns heterogemini sulfobetaines and their derivatives, which seem to be a new generation of surfactants. The procedure for the synthesis of *N*-methyl-*N*-[6-(*N*-alkyl-*N*-methylamine)hexyl]propylammonium 3-sulfate and their chloride salts, where alkyl represents the hydrocarbon chain lengths of 12, 14 and 16, in a two-step reaction is described. Moreover, their surface properties such as equilibrium and dynamic surface tension are investigated. The value of CMC, surface pressure at the CMC (Π_CMC_), p20 and standard free energy of micellization ($\Delta G_{m}^{0}$) are calculated.

## Experimental Methods

### Materials

*N*,*N*′-Dimethyl-1,6-hexanediamine, alkyl bromide and 1,3-propanesultone were purchased from Sigma–Aldrich. Potassium carbonate and acetone were obtained from Chempur; acetonitrile was purchased from POCh SA. All compounds were AR quality and they were used without further purification.

All the surfactants solutions were prepared with the use of water from the PURELAB Classic, Elga with resistivity 18.2 MΩ cm.

### Synthesis Procedures

The first step was to synthesise *N*,*N*′-dialkyl**-** *N*,*N*′-dimethyl-1,6-diaminohexane. For this purpose *N*,*N*′-dimethyl-1,6-hexanediamine (0.02 mol, 3 g) was reacted with alkyl bromide (0.04 mol) in the presence of potassium carbonate (0.04 mol, 5.74 g) in 80 cm^3^ acetonitrile. The reaction mixture was refluxed for several hours (the reaction time was in the range 18–40 h depending on the length of the alkyl bromide chain). The resultant white sediment was recrystallized from a mixture of acetone and acetonitrile (9:1, v/v). This procedure afforded amines with two dodecyl, tetradecyl and hexadecyl groups. The required amine (0.005 mol) was then reacted with 1,3-propanesultone (0.01 mol, 1.32 g) in anhydrous acetone at the molar ratio 1:2. The reaction mixture was refluxed for more than 19 h. The solvent was evaporated under reduced pressure and the obtained solid was recrystallized from a mixture of ethyl acetate and methanol (9:1, v/v) to afford the hexamethyl-1,6-bis-(*N*-alkyl-*N*-methylammonio-*N*-propylsulfonate) homogemini surfactants [11]. Subsequently, these compounds reacted with a suitable *N*,*N*′-dialkyl**-** *N*,*N*′-dimethyl-1,6-diaminohexane in anhydrous acetone at the molar ratio 1:1. The reaction mixture was refluxed for several hours and the product was filtered off and purified by recrystallization from a mixture of ethyl acetate and methanol (9:1, v/v). White solids of *N*-methyl-*N*-[6-(*N*-alkyl-*N*-methylamine)hexyl]propylammonium 3-sulfate were obtained.

Moreover, *N*-alkyl-*N*-methyl-*N*-(3-sulfopropyl)-6-(*N*-alkyl-*N*-methylamino)hexylammonium chlorides were synthesised. In the first step of the synthesis, *N*-methyl-*N*-[6-(*N*-alkyl-*N*-methylamine)hexyl]propylammonium 3-sulfate and 0.1 M hydrochloride acid were heated to boiling point. Then the solvent was evaporated and the solid obtained was crystallized from a mixture of ethyl acetate and methanol (9:1, v/v).

**Fig. 1:**
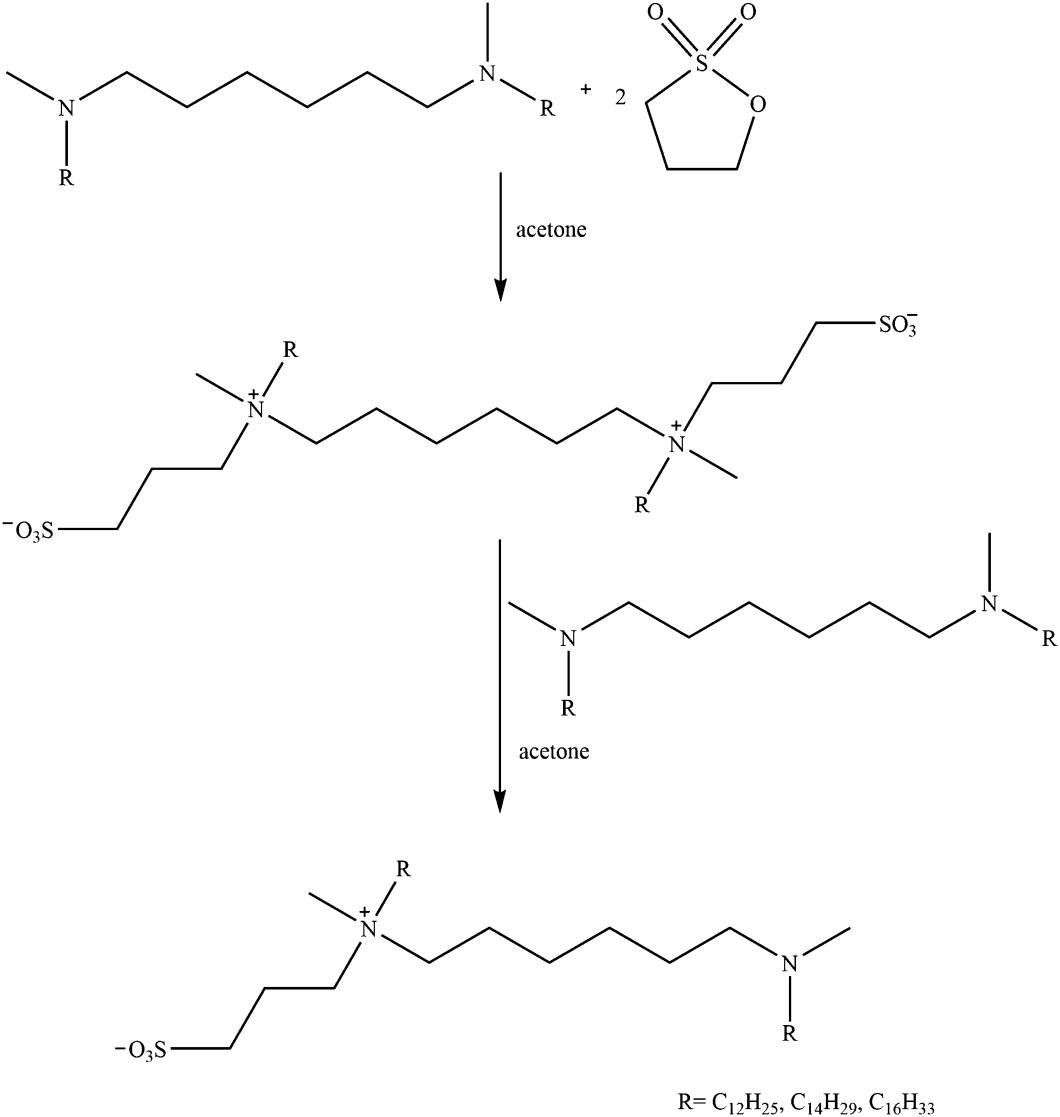
Synthesis route of novel zwitterionic heterogemini surfactants

Figure 1 shows the synthesis route of novel zwitterionic heterogemini surfactants.

All newly obtained surfactants are listed in Table 1. They were characterised by ^1^H NMR and ^13^C NMR (Varian Merkury, Gemini+300VT), IR (Perkin Elmer) and elemental analysis (Elementar Analyser Vario EL III). Melting points were determined with a Boetius apparatus.

**Table 1:** Surfactants synthesised in the study and abbreviations used in this work

No.	Surfactant name	Formula	Abbreviation
1	*N*-Methyl-*N*-[6-(*N*-dodecyl-*N*-methylamine)hexyl]propylammonium 3-sulfate	C_35_H_74_N_2_O_3_S	DMH-12C3S
2	*N*-Methyl-*N*-[6-(*N*-methyl-*N*-tetradecylamine)hexyl]propylammonium 3-sulfate	C_39_H_82_N_2_O_3_S	DMH-14C3S
3	*N*-Methyl-*N*-[6-(*N*-hexadecyl-*N*-methylamine)hexyl]propylammonium 3-sulfate	C_43_H_90_N_2_O_3_S	DMH-16C3S
4	*N*-Dodecyl-*N*-methyl-*N*-(3-sulfopropyl)-6-(*N*-dodecyl-*N*-methylamino)hexylammonium chloride	C_35_H_75_N_2_O_3_SCl	DMH-12C3S·HCl
5	*N*-Methyl-*N*-(3-sulfopropyl)-*N*-tetradecyl-[6-(*N*-methyl-*N*-tetradecyl)amino]hexylammonium chloride	C_39_H_83_N_2_O_3_SCl	DMH-14C3S·HCl
6	*N*-Hexadecyl-*N*-methyl-*N*-(3-sulfopropyl)-6-(*N*-methyl-*N*-hexadecylamino)hexylammonium chloride	C_43_H_91_N_2_O_3_SCl	DMH-16C3S·HCl

### Synthesis of *N*-Alkyl-*N*-methyl-*N*-(3-sulfopropyl)-6-(*N*-alkyl-*N*-methylamino)hexylammonium chloride

#### *N*-Methyl-*N*-[6-(*N*-dodecyl-*N*-methylamine)hexyl]propylammonium 3-sulfate (1)

Yield 52.1 %; m.p. 198 °C; ^1^H NMR (CDCl_3_) *δ* = 0.88 (*J* = 7, t, 6H, CH_3_), 1.26 (m, 40H, CH_2_), 1.46 (*J* = 6.8, d, 4H, CH_2_), 1.68 (m, 2H, CH_2_), 2.22 (m, 4H, CH_2_N), 2.32 (m, 4H, CH_2_), 2.86 (s, 2H, CH_2_N^+^), 3.17 (m, 4H, CH_2_N^+^), 3.24 (*J* = 18, t, 3H, CH_3_), 3.39 (m, 3H, CH_3_N^+^), 3.66 (m, 2H, CH_2_SO_3_^−^); ^13^C NMR (CDCl_3_) *δ* = 14.1 (2 × CH_3_), 19.0 (CH_2_), 20.8 (2 × CH_2_), 22.6 (2 × CH_2_), 24.7 (CH_2_), 26.4 (CH_2_), 27.1 (CH_2_), 27.5 (CH_2_), 29.3 (2 × CH_2_), 29.6 (4 × CH_2_), 31.8 (4 × CH_2_), 42.2 (2 × CH_2_), 47.6 (CH_3_N), 48.4 (CH_3_N^+^), 57.8 (CH_2_SO_3_^−^), 60.2 (2 × CH_2_N), 61.5 (CH_2_N^+^), 61.9 (2 × CH_2_N^+^). IR: 2,920, 2,852, 1,470, 1,188, 1,034 cm^−1^. Elemental analysis for DMH-12C3S: Found: C = 69.1 %, H = 12.25 %, N = 4.38 %, S = 4.89 %; Calculated: C = 69.77 %, H = 12.29 %, N = 4.65 %, S = 5.23 %.

#### *N*-Methyl-*N*-[6-(*N*-methyl-*N*-tetradecylamine)hexyl]propylammonium 3-sulfate (2)

Yield 77.8 %; m.p. 218 °C; ^1^H NMR (CDCl_3_) *δ* = 0.88 (*J* = 6.9, t, 6H, CH_3_), 1.26 (m, 48H, CH_2_), 1.45 (*J* = 6.3, d, 4H, CH_2_), 1.67 (m, 2H, CH_2_), 2.20 (m, 4H, CH_2_N), 2.30 (m, 4H, CH_2_), 2.87 (s, 2H, CH_2_N^+^), 3.16 (m, 4H, CH_2_N^+^), 3.26 (*J* = 15.2, t, 3H, CH_3_), 3.40 (m, 3H, CH_3_N^+^), 3.64 (m, 2H, CH_2_SO_3_^−^); ^13^C NMR (CDCl_3_) *δ* = 14.1 (2 × CH_3_), 19.0 (CH_2_), 21.0 (2 × CH_2_), 22.6 (2 × CH_2_), 24.8 (CH_2_), 26.4 (CH_2_), 27.3 (CH_2_), 27.6 (CH_2_), 29.3 (2 × CH_2_), 29.6 (6 × CH_2_), 31.9 (4 × CH_2_), 42.3 (2 × CH_2_), 47.6 (CH_3_N), 48.4 (CH_3_N^+^), 57.9 (CH_2_SO_3_^−^), 60.3 (2 × CH_2_N), 61.5 (CH_2_N^+^), 61.9 (2 × CH_2_N^+^). IR: 2,917, 2,850, 1,471, 1,187, 1,035 cm^−1^. Elemental analysis for DMH-14C3S: Found: C = 69.3 %, H = 13.65 %, N = 4.27 %, S = 4.77 %; Calculated: C = 71.1 %, H = 12.46 %, N = 4.26 %, S = 4.86 %.

#### *N*-Methyl-*N*-[6-(*N*-hexadecyl-*N*-methylamine)hexyl]propylammonium 3-sulfate (3)

Yield 72 %; m.p.194 °C; ^1^H NMR (CDCl_3_) *δ* = 0.88 (*J* = 6.7, t, 6H, CH_3_), 1.26 (m, 58H, CH_2_), 1.47 (*J* = 7.6, d, 4H, CH_2_), 1.67 (m, 2H, CH_2_), 2.21 (m, 4H, CH_2_N), 2.30 (m, 4H, CH_2_), 2.87 (s, 2H, CH_2_), 3.28 (m, 4H, CH_2_N^+^), 3.17 (*J* = 4.8, t, 3H, CH_3_), 3.39 (m, 3H, CH_3_N^+^), 3.64 (m, 2H, CH_2_SO_3_^−^); ^13^C NMR (CDCl_3_) *δ* = 14.1 (2 × CH_3_), 19.0 (CH_2_), 20.9 (2 × CH_2_), 22.6 (2 × CH_2_), 24.7 (CH_2_), 26.5 (CH_2_), 27.2 (CH_2_), 27.6 (CH_2_), 29.3 (2 × CH_2_), 29.6 (8 × CH_2_), 31.9 (4 × CH_2_), 42.3 (2 × CH_2_), 47.6 (CH_3_N), 48.4 (CH_3_N^+^), 57.9 (CH_2_SO_3_^−^), 60.3 (2 × CH_2_N), 61.4 (CH_2_N^+^), 62.0 (2 × CH_2_N^+^). IR: 2,916, 2,850, 1,472, 1,189, 1,035 cm^−1^. Elemental analysis for DMH-16C3S: Found: C = 69.35 %, H = 14.04 %, N = 3.34 %, S = 4.26 %; Calculated: C = 72.27 %, H = 12.6 %, N = 3.92 %, S = 4.48 %; Calculated for DMH-16C3S+2H_2_O: C = 68.8 %, H = 12.53 %, N = 3.73 %, S = 4.27 %.

#### *N*-Dodecyl-*N*-methyl-*N*-(3-sulfopropyl)-6-(*N*-dodecyl-*N*-methylamino)hexylammonium chloride (4)

Yield 74.9 %; m.p. 196 °C; IR: 2,921, 2,853, 2,615-2,511, 1,468, 1,198, 1,036 cm^−1^. Elemental analysis for DMH-12C3S·HCl: Found: C = 61.28 %, H = 10.80 %, N = 3.69 %, S = 5.03 %; Calculated: C = 65.78 %, H = 11.75 %, N = 4.39 %, S = 5.01 %.

#### *N*-Methyl-*N*-(3-sulfopropyl)-*N*-tetradecyl-[6-(*N*-methyl-*N*-tetradecyl)amino]hexylammonium chloride (5)

Yield 87.8 %; m.p. 188 °C; IR: 2,920, 2,852, 2,601–2,503, 1,469, 1,201, 1,035 cm^−1^. Elemental analysis for DMH-14C3S·HCl: Found: C = 64.64 %, H = 10.93 %, N = 3.75 %, S = 4.35 %; Calculated: C = 67.39 %, H = 11.95 %, N = 4.03 %, S = 4.61 %.

#### *N*-Hexadecyl-*N*-methyl-*N*-(3-sulfopropyl)-6-(*N*-methyl-*N*-hexadecylamino)hexylammonium chloride (6)

Yield 86.1 %; m.p. 194 °C; IR: 2,914, 2,849, 2,624–2,511, 1,469, 1,206, 1,036 cm^−1^. Elemental analysis for DMH-16C3S·HCl: Found: C = 63.98 %, H = 12.27 %, N = 3.06 %, S = 4.10 %; Calculated: C = 68.75 %, H = 12.13 %, N = 3.73 %, *S* = 4.26 %.

The yield was calculated as the ratio of the chemical reaction product to the mass of the product calculated from the chemical reaction equation on the basis of stoichiometric coefficients used in the equation and amounts of substrates used.

### Measurements

#### Equilibrium Surface Tension

The surface tension of the aqueous solutions of surfactants synthesised was measured at a constant temperature by the drop shape method with a Tracker (I.T. Concept, France) tensiometer. The optical tensiometer captures an image of an air drop immersed in an aqueous solution of surfactant and records the drop shape as a function of time. The drop shape is determined by the surface tension of the liquid, gravity and the density difference between the air and surrounding medium. The drop image is analysed with a profile fitting method in order to determine the contact angle and the surface tension.

Additional physicochemical analyses on the basis of the surface tension data were carried out using the Szyszkowski equation [12].

#### Dynamic Surface Tension

The dynamic surface tension was measured using a Sita bubble pressure tensiometer science line t60. The bubble lifetime, from 30 ms to 60 s (with resolution 1 ms), permits dynamic and semistatic measurements of surface tension.

## Results and Discussion

### Surface Tension and Critical Concentration of *N*-Alkyl-*N*-methyl-*N*-(3-sulfopropyl)-6-(*N*-alkyl-*N*-methylamino)hexylammonium chloride

**Fig. 2:**
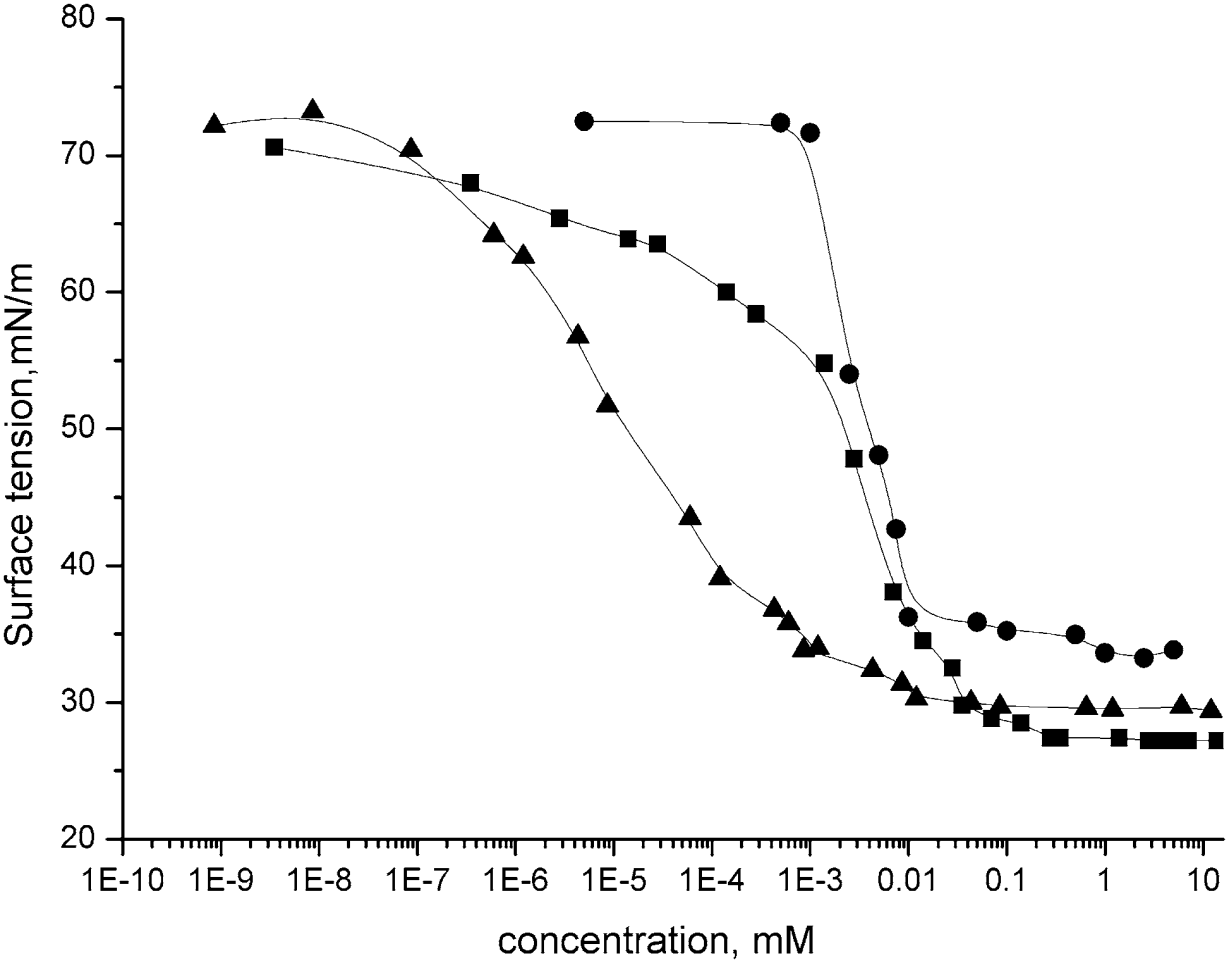
Surface tension isotherms for *closed triangle* DMH-12C3S·HCl, *closed circle* DMH-14C3S·HCl, *closed square* DMH-16C3S·HCl air/water system, concentration *c* in mmol/dm^3^, temperature 21 °C

The results of surface tension measurements for aqueous solutions of *N*-alkyl-*N*-methyl-*N*-(3-sulfopropyl)-6-(*N*-alkyl-*N*-methylamino)hexylammonium chloride are shown in Fig. 2. The CMC and surface tensions at the CMC were determined from the inflection point on the curves of the surface tension versus logarithm of concentration and presented in Table 2. The results indicate that the values of CMC decrease with increasing chain length of the synthesised chlorides. This phenomenon could be explained by the increasing hydrophobicity of the alkyl moiety.

**Table 2:** Surface properties of surfactants

Abbreviation	CMC (mM)	*γ*_CMC_ (mN/m)	p20	Π_CMC_ (mN/m)	$\Delta G_{m}^{0}$ (kJ/mol)
DMH-12C3S·HCl	0.0345	27.77	2.72	44.47	−70.08
DMH-14C3S·HCl	0.0093	36.28	2.35	36.77	−76.49
DMH-16C3S·HCl	0.0011	30.45	4.60	41.79	−87.18

**Fig. 3:**
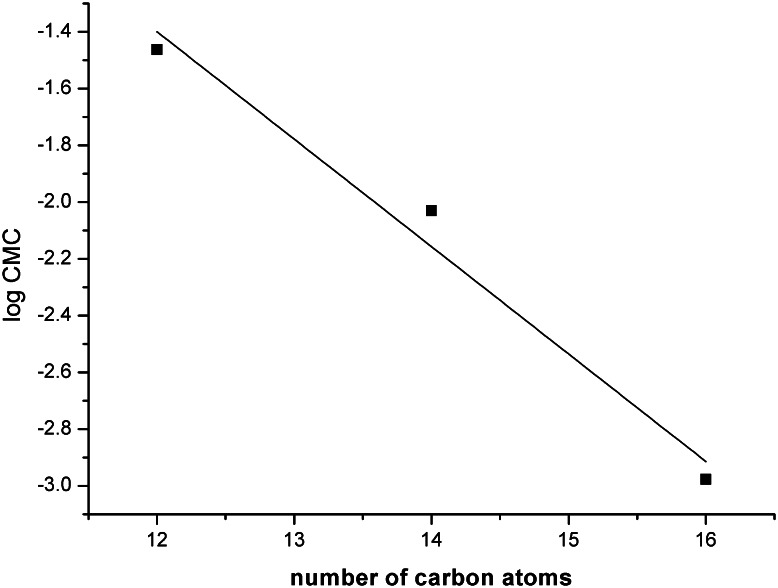
Dependence between CMC and alkyl length chain of *N*-alkyl-*N*-methyl-*N*-(3-sulfopropyl)-*N*-[6-(*N*′-alkyl-*N*′-methylaminohexyl)]ammonium chlorides

For homologues straight-chain surfactants, a relation between the number of carbon atoms in the hydrophobic chain and the CMC can be written in the form

1 $$\log \;({\text{CMC}})\; = \;A\; - \;B\; \times \;n$$

where *A* is a constant for a particular ionic head at a given temperature and *B* is close to 0.3 at 35 °C for the conventional anionic and cationic surfactants and 0.5 for nonionic and zwitterionic ones [2]. The relationship between hydrocarbon chain length of *N*-alkyl-*N*-methyl-*N*-(3-sulfopropyl)-6-(*N*-alkyl-*N*-methylamino)hexylammonium chloride and CMC is shown in Fig. 3. The value of *B* is 0.37. This value is smaller than that for other gemini surfactants (0.4–0.46) [2]. This means that the decrease in the CMC values with increasing chain length for the surfactants studied is smaller. The excellent micelle-forming ability at low concentration of the surfactants studied could be explained by the driving forces following from the interaction between hydrocarbon chains connected by a short spacer chain as well as by a decline of electrostatic repulsion between ammonium and sulfonate headgroups.

On the basis of the value of surface pressure determined at CMC (Π_CMC_), p20 and standard free energy of micellization, the values of ($\Delta G_{m}^{0}$) were calculated and listed in Table 2. The value of p20 informs about the efficiency of adsorption of the surfactant at the air–water interface. The higher the value of p20, the greater the tendency of the surfactant to adsorb at the air–water interface, relative to its tendency to form micelles, and the more efficient its reduction of the surface tension [8]. From among the chlorides synthesised the lowest p20 value was obtained for DMH-14C3S·HCl. The local minimum in the dependence of p20 and the chain length observed for DMH-14C3S·HCl is typical of this homologue of zwitterionic surfactants. A similar relationship has been observed for zwitterionic heterogemini surfactants containing ammonium and carboxylate headgroups [2]. The authors of this paper have observed that the p20 values increase with increasing hydrocarbon chain length up to 12, and the high homologue with chain length of 14 deviates from this trend, as it is characterised by a smaller value of p20. This phenomenon could be attributed to premicellar aggregation leading to longer chain lengths.

The values of CMC could be used for calculation of the molar free enthalpy of the micellization process. Analysis of the structures of the chlorides obtained showed that the micellar association process of these compounds is best described by the pseudophase model, according to the following equation:

2 $$m{\text{D}}^{ + } + \, m{\text{X}}^{ - } \to \, M$$

The model assumes that the micelle (M) is formed by the surfactant ions (D^+^) and bonded with the counterions (X^−^). From the thermodynamic point of view, this process can be described by the following equation:

3 $$\Delta G_{\text{m}}^{ 0} \; = \;2{\text{RTl}}nX_{\text{CMC}}$$

where *R* is the gas constant, *T* is the absolute temperature and *X*_CMC_ is the molar fraction of surfactant [13].

The values of $\Delta G_{{_{m} }}^{{^{0} }}$ of the heterogemini surfactants studied are negative (Table 2), indicating that the surfactants show great ability to form micelles in aqueous solution. The standard free energy of micellization becomes large (more negative) with increasing hydrocarbon chain length. This indicates that there is no stereo inhibition of two longer hydrocarbon chains of heterogemini surfactant, as it forms micelles. The same effect was found for alkylsulfopropanebetaines [14]. The free energy of micellization was found to be −12 and −23.5 kJ/mol for octylsulfopropanebetaine and dodecyl derivative, respectively. The more negative values of $\Delta G_{m}^{0}$, for longer alkyl chain in the sulfobetaine molecule, suggest that the hydrophobic interactions, which are much stronger for larger derivatives, favour micellar aggregation.

Additional physicochemical analyses on the basis of the surface tension data were carried out as follows. First, the surface tension data were fitted by Szyszkowski’s equation. The values of the adsorption coefficients of the Szyszkowski isotherm (*A*_sz_ and *B*_sz_) allowed the calculation of the surface excess at the saturated interface (*Γ*_∞_), the minimum molecular area in the adsorption layer at the saturated interface (*A*_min_) and the Gibbs free energy of adsorption (Δ*G*_ads_) according to the following equations [12]:

4 $$\varGamma_{\infty } \; = \;\frac{{B_{\text{Sz}} \gamma_{0} }}{\text{RT}}$$

5 $$A_{\hbox{min} } \; = \;\frac{1}{{\varGamma_{\infty } N_{\text{A}} }}$$

6 $$\Delta G_{\text{ads}}^{\text{Sz}} \; = \;{\text{RT}}\ln (A_{\hbox{min} } )$$

where *γ*_0_, *N*_A_, *R* and *T* stand for interfacial tension for concentration *c* = 0, the Avogadro constant, gas constant and temperature, respectively.

All these parameters are listed in Table 3. Analysis of these data allowed us to conclude that it is impossible to unambiguously state that the minimum value of the area occupied by a single adsorbed molecule increases with increasing elongation of the alkyl chains. The lack of such a relation has been described in the literature for heterogemini sulfobetaines [four kinds of sulfobutane betaines H-(CH_2_)_*n*_N^+^(CH_3_)_2_(CH_2_)_4_SO_3_^−^ with *n* = 12, 14, 16, 18] [4] and for a series of alkylbetaine zwitterionic gemini surfactants, 1,2-bis(*N*-methyl-*N*-carboxymethyl-alkylammonium)ethane (CnAb, *n* = 8, 10, 12, 14) [10]. In addition to the above, there have been no reported relationships between the surface excess at the saturated interface and the length of the alkyl chain for sulfobetaines *N*,*N*-dimethyl-*N*-{2-[*N*′-methyl-*N*′-(3-sulfopropyl)alkylammonium]ethyl}-1-alkylammonium bromides [2C(*n*)AmSb, with *n* = 8, 10, 12, 14] [7]. Also for the chloride derivatives of the heterogemini sulfobetaines studied, there is no relationship between the surface excess at the saturated interface and the length of the alkyl chain. For the studied systems, however, there is a relationship involving the decline in the value of free energy of adsorption accompanying the increasing elongation of the alkyl chains. The values of Δ*G*_ads_, similarly to those of the standard free energy of micellization, are negative, indicating that the surfactants studied have a great ability to adsorb at the air–water interface. Moreover, the absolute values of $\Delta G_{m}^{0}$ are higher than that of Δ*G*_ads_, indicating that the micellization is preferred to the adsorption process.

**Table 3:** Adsorption parameters of surfactants in water/air systems

Abbreviation	*B*_Sz_ × 10^2^	*A*_Sz_ (mol/dm^3^)	*Γ* ^∞^ × 10^6^ (mol/m^2^)	Δ*G*_ads_ (kJ/mol)	*A*_min_ (m^2^)
DMH-12C3S·HCl	7.13 × 10^−2^	1.4 × 10^−8^	2.1 × 10^−6^	−44.2	7.91 × 10^−19^
DMH-14C3S·HCl	3.58 × 10^−2^	6.13 × 10^−10^	1.07 × 10^−6^	−51.9	1.56 × 10^−18^
DMH-16C3S·HCl	5.25 × 10^−2^	5.62 × 10^−11^	1.55 × 10^−6^	−57.7	1.07 × 10^−18^

It should be noted that adsorption properties of gemini surfactants often are different from those of other surfactants. There are some examples in the literature that the values of *A*_min_ increase with increasing number of carbons in the hydrophobic tail, suggesting that gemini surfactants with shorter hydrophobic tails have higher packing densities at the air–water surface. This phenomenon could be explained by the longer hydrophobic chains being more prone to curl [15–17].

### Dynamic Surface Tension of *N*-Alkyl-*N*-methyl-*N*-(3-sulfopropyl)-6-(*N*-alkyl-*N*-methylamino)hexylammonium chloride

**Fig. 4:**
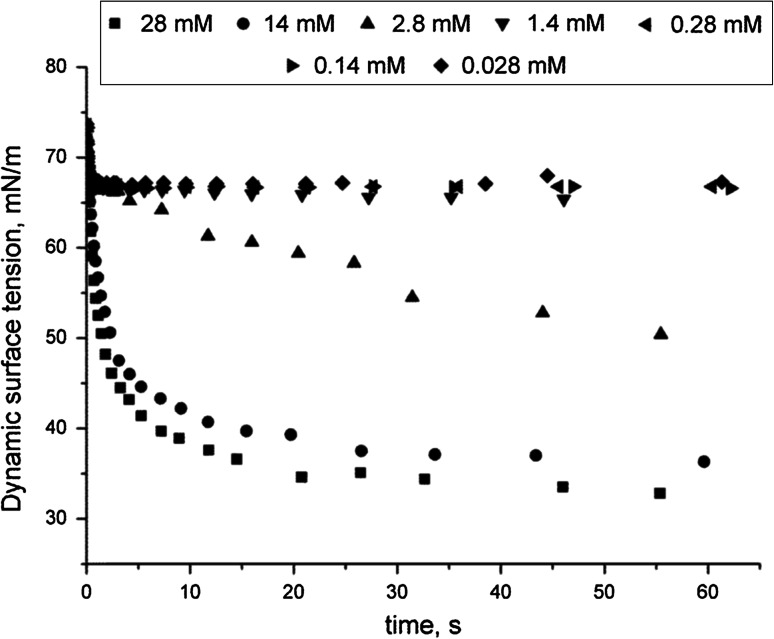
Dynamic interfacial tension as a function of time for DMH-12C3S·HCl in the water–air system

The dynamic surface tension measurements of surfactant aqueous solutions were performed by the maximum bubble pressure technique. Figure 4 shows the exemplary time dependence of the dynamic surface tension for DMH-12C3S·HCl at concentrations below and above the CMC. For the surfactant in a low concentration, in the bulk phase, the values of dynamic interfacial tension reach the equilibrium value in a longer time than in the systems with the surfactant at a higher concentration. A similar relationship was observed by other researchers [18, 19]. The higher the concentrations of heterogemini surfactants, the faster the adsorptions at the air–water interface. At concentrations above the CMC, the values of reduced dynamic surface tension are nearly close to the equilibrium ones, suggesting a fast adsorption process of DMH-12C3S·HCl. A similar relationship was observed for surfactants studied with chain lengths of 14 and 16.

The adsorption of surfactant molecules at the interface can consist of two different processes: diffusion from the bulk phase to the sublayer and transfer from the sublayer to the interface without diffusion. Depending upon the relative contribution of both processes considered, the adsorption can take place in diffusion, kinetic or mixed regions, which means that the adsorption can be controlled by either diffusion from the bulk to the sublayer or transfer from the sublayer to the interface or both these steps.

In order to check if the adsorption process is diffusion controlled or not, one can use the approximation of the general diffusion equation of Ward and Tordai [20]. For neutral molecules two approximations can be applied [21]:

- A short-time approximation (for the beginning of the adsorption process):

7 $$\gamma_{t \to 0} \; = \;\gamma_{0} \; - \;2n{\text{RT}}c_{0} \sqrt {\frac{Dt}{\pi }}$$

- A long-time approximation (when the adsorption process is near equilibrium):

8 $$\gamma_{t \to \infty } \; = \;\gamma_{\text{eq}} \; + \frac{{n{\text{RT}}\varGamma_{\text{eq}}^{2} }}{c}\;\sqrt {\frac{\pi }{4Dt}}$$

The parameters *c*, *Γ* and *D* represent the bulk concentration, equilibrium surface excess and monomer diffusion coefficient of the surfactant, respectively.

The values of diffusion coefficients (*D*) for single surfactants can thus be obtained from dynamic interfacial tension measurements depending on the adsorption process being in the initial stage or near equilibrium. Most of the literature data on dynamic surface tension of surfactant solutions are linearized when plotted as *t* ^1/2^ or *t* ^**−**1/2^ as suggested by Eqs. (7) and (8). Nonetheless, it is still not clear whether the adsorption is purely diffusion controlled over the entire time range, or if these equations can really be used to predict *y*(*t*).

**Fig. 5:**
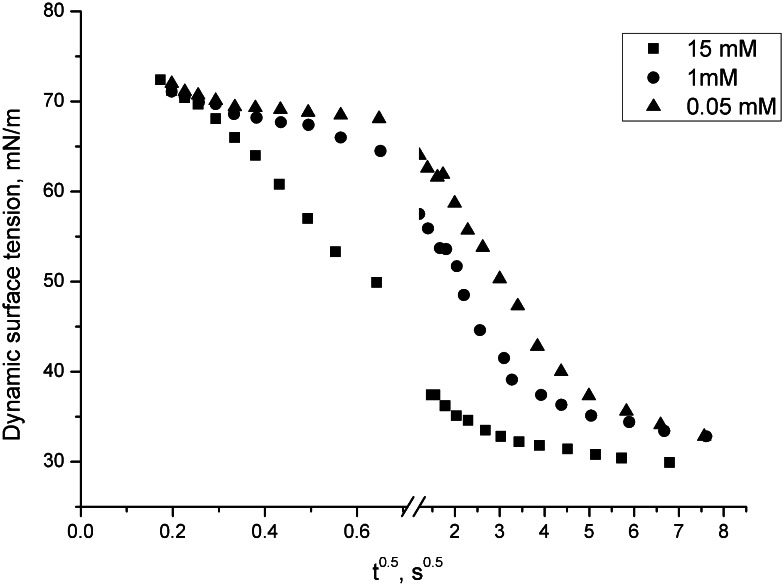
Dynamic surface tension as a function square root of the age of the interface for DMH-14C3S·HCl in the water–air system; concentration 15, 1 and 0.05 mM

**Fig. 6:**
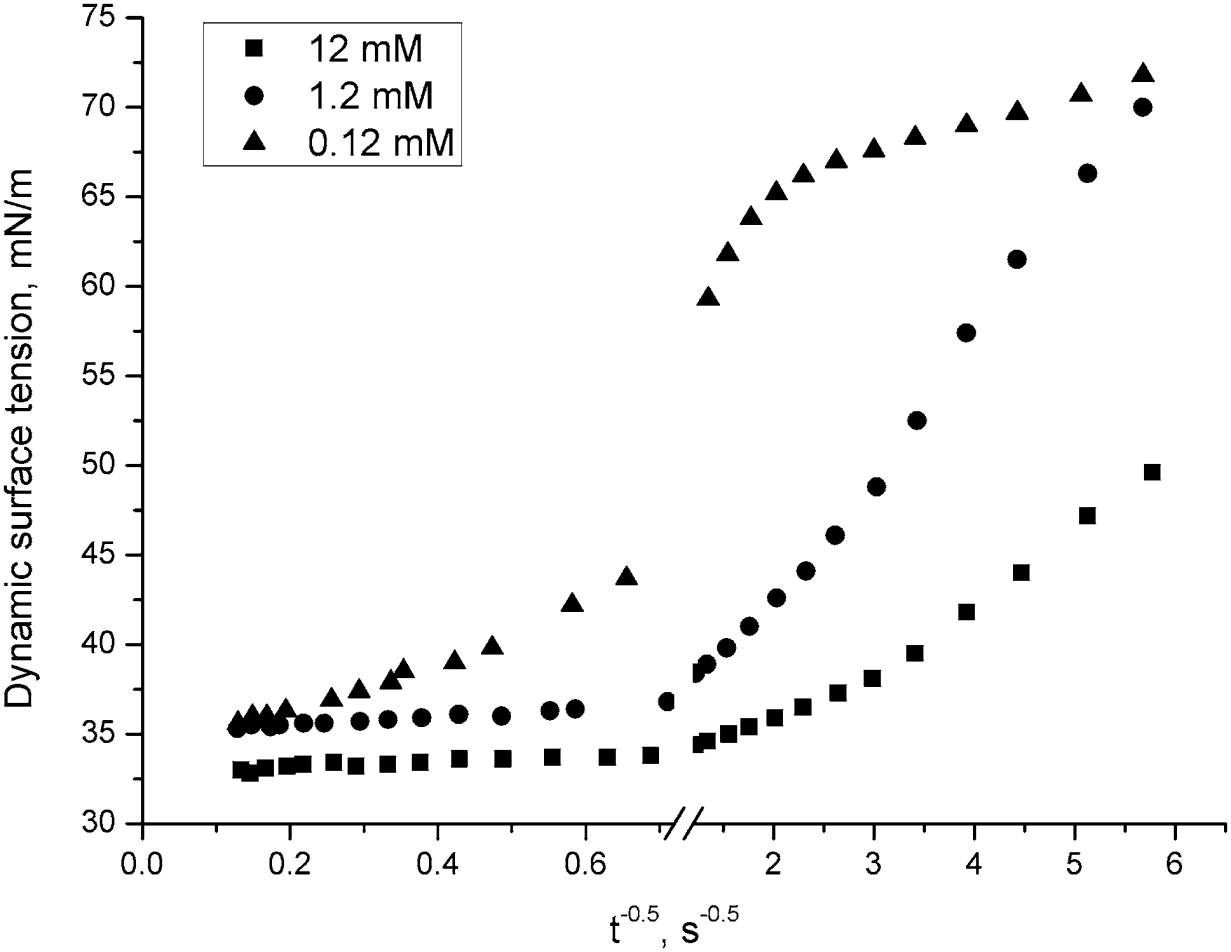
Dynamic surface tension as a function of reciprocal of square root of the age of the interface for DMH-16C3S·HCl in the water–air system; concentration 12, 1.2 and 0.12 mM

For the DMH-14C3S·HCl and DMH-16C3S·HCl at different concentrations, the plots of dynamic surface tension versus *t* ^1/2^ and *t* ^−1/2^ are shown in Figs. 5 and 6, respectively. These plots show a linear behaviour over the shorter time scales (low values of *t* ^1/2^) and the longer time scales (low values of *t* ^−1/2^). The straight lines obtained, which are representative of all the other systems studied, indicate that the adsorption process of surfactants studied in the water–air system is diffusion controlled and the diffusion coefficients can be calculated according to Eqs. (7) and (8), respectively. They are shown in Table 4.

**Table 4:** Values of diffusion coefficient for surfactants studied

Compounds	*c* (mM)	Diffusion coefficients *D* (m^2^/s)	
		*D*_*t*→0_	*D*_*t*→∞_
DMH-12C3S·HCl	28	7.25 × 10^−14^	6.53 × 10^−16^
	14	2.47 × 10^−13^	1.75 × 10^−16^
	2.8	2.93 × 10^−11^	1.97 × 10^−14^
	1.4	2.01 × 10^−11^	6.52 × 10^−12^
DMH-14C3S·HCl	5	5.03 × 10^−13^	1.43 × 10^−16^
	2.5	2.52 × 10^−12^	3.99 × 10^−16^
	0.1	8.92 × 10^−10^	6.55 × 10^−12^
	0.05	2.72 × 10^−09^	2.79 × 10^−9^
DMH-16C3S·HCl	12	2.21 × 10^−12^	8.48 × 10^−14^
	6	1.69 × 10^−11^	3.20 × 10^−13^
	0.6	3.38 × 10^−9^	4.77 × 10^−12^
	0.012	1.31 × 10^−7^	2.73 × 10^−11^

The values of the diffusion coefficient of *N*-alkyl-*N*-methyl-*N*-(3-sulfopropyl)-6-(*N*-alkyl-*N*-methylamino)hexylammonium chloride tend to decrease as the concentration is increased. Similar relations between the values of diffusion coefficient and the bulk surfactant concentration have also been observed by other authors in studies of other surfactants in hydrocarbon–water systems [22–26] and air–water once [27–29]. The lower values of diffusion coefficients for higher surfactant concentrations could be explained by increasing concentration of aggregates in more concentrated solutions because of the association process [30]. On the other hand, with increasing bulk concentration the mass transport could change as a result of modification of the adsorption process from pure diffusion to activation–diffusion [30, 31]. Lin et al. [24, 32], in studies of the adsorption process of polyoxyethylene alcohols in air–water systems, concluded that the controlling mechanism for mass transfer can change as a function of bulk concentration from diffusion to mixed kinetic-diffusion control. From examining the changes in diffusion coefficient with the surfactant concentration, those authors suggested a diffusion-controlled mechanism at dilute bulk concentrations, where the equilibrium surface coverage is low, and mixed kinetic-diffusion control as the bulk concentration grows and the equilibrium surface coverage is increased.

The diffusion coefficient of surfactants obtained by the short-time approximation model is not consistent with that in the long-time behaviour. It could be explained by uncertainty of the surface excess concentration obtained from the surface tension measurement and the presence of an adsorption barrier [33]. Moreover, the values of diffusion coefficient estimated according to the long-time approximation model are too small, indicating the diffusion of the solute molecules to the subsurface and adsorption of the solute from the subsurface to the surface [34].

The dynamic surface tension of micellar surfactant solutions depends on the diffusion rates of monomers and micelles, and on the dissociation/dissolution process of the micelles as this represents an additional source of the transport of surfactant molecules. The micelle dissociation constants from dynamic surface tension data could be calculated from the equation proposed by Frese et al. [35]:

9 $$\frac{{[dy/dt^{ - 1/2} ]_{\text{CMC}} }}{{[dy/dt^{ - 1} ]_{{c > {\text{CMC}}}} }}\; = \;\alpha \left( {\frac{{k_{2} \pi }}{2}} \right)^{1/2} \; = \;\alpha \left( {\frac{\pi }{4}} \right)^{1/2} \;\tau_{2}^{ - 1/2}$$

here *k*_2_ is the micelle dissociation rate constant, which is identical to the inverse relaxation time of the slow micelle kinetics process, *k*_2_ = *τ* ^−1/2^, and *α* is the relative concentration of monomers at *c* > CMC with respect to that at *c* *=* CMC. The derivative d*γ*/d*t* ^−1*/*2^ is determined from the *γ* dependence on *t* ^−1*/*2^ for *t* → ∞ at *c* *=* CMC, and the derivative d*γ*/d*t* ^−1^ is determined from the *γ* dependence on *t* ^−1^ for *t* → ∞ at any concentration above CMC. According to the suggestion from the work cited, *α* = 1 was assumed.

In a typical surfactant solution above CMC there are monomers and aggregates (micelles) in a distribution around the average aggregation number.

Moreover, the time in which monomers are present in the micelle structure, called the micelle lifetime (*T*_m_), was also estimated according to the following equation [13]:

10 $$T_{m} \; = \;m\tau$$

The value of *τ* is the reciprocal of the dissociation constant *k*_2_ and the relaxation time corresponding to the free micelle formation step. In the above formula, *m* indicates the average number of micelles aggregation. However there are no literature data on the average aggregation number of micelles of the compounds studied. Therefore, the values for the surfactants of the same alkyl chain length (55 for C12, 40 for C14 and 80 for C16) [13] were assumed as the average aggregation numbers of micelles. The estimated values of micelle dissociation rate constant (*k*_2_) and micelle lifetime (*T*_m_) are presented in Table 5.

**Table 5:** Dissociation kinetics of *N*-alkyl-*N*-methyl-*N*-(3-sulfopropyl)-6-(*N*-alkyl-*N*-methylamino)hexylammonium chloride micelles

*c* (mM)	d*γ*/d*t* ^−1/2^ [(mM/m)s^1/2^]	d*γ*/d*t* ^−1^ [(mM/m)s]	*k*_2_ (s^−1^)	*T*_m_ (s)
DMH-12C3S·HCl				
28	28.61	56.30	7.91 × 10^−5^	1,011,894.49
14	28.41	49.87	1.01 × 10^−4^	793,772.34
2.8	14.39	14.68	1.16 × 10^−3^	68,758.17
1.4	5.32	11.15	2.01 × 10^−3^	39,710.90
0.028	0.44	0.80	3.91 × 10^−1^	204.62
DMH-14C3S·HCl				
5	41.46	65.65	9.38 × 10^−1^	2,901.95
2.5	41.03	42.13	6.02 × 10^−1^	2,872.21
0.1	9.41	11.26	1.61 × 10^−1^	658.88
0.05	0.91	1.16	1.66 × 10^−2^	63.88
0.0025	0.70	1.23	1.76 × 10^−2^	49.13
0.001	0.59	0.77	1.10 × 10^−2^	41.22
DMH-16C3S·HCl				
12	4.15	10.43	9.22 × 10^−3^	5,967.75
6	2.84	5.78	3.00 × 10^−2^	1,830.73
1.2	2.32	4.51	4.93 × 10^−2^	1,116.08
0.6	3.94	4.13	5.87 × 10^−2^	937.64
0.12	9.38	2.49	1.61 × 10^−1^	340.69
0.06	6.82	1.89	2.81 × 10^−1^	195.87
0.012	3.09	1.84	2.96 × 10^−2^	185.55

The values of the micelle lifetime are greater for the compounds substituted with longer alkyl chains. The time in which the monomers are present in the micelle structure for the surfactants with 12, 14 or 16 carbons in the chain ranges accordingly from 3 to 16,865, 0.7 to 48 and 3 to 99 min, respectively. The lifetime of micelles decreases with decreasing concentration of the surfactant in the solution. The compounds characterized by an extended structure need much less time to locate into the micelles than compounds of a smaller particle size. Moreover, the time for the exit process from the micelle structures by molecules of smaller compounds is longer.

## Conclusions

*N*-Methyl-*N*-[6-(*N*-alkyl-*N*-methylamine)hexyl]propylammonium 3-sulfate and its chloride salt with hydrocarbon chain lengths of 12, 14 and 16 were synthesised, and their surface-active properties were characterized by measuring the equilibrium and dynamic surface tension at the water–air interface. The results show that the heterogemini sulfobetaines with longer hydrophobic chains have a lower CMC value. Moreover, it was found that the adsorption properties and micelle lifetime of these compounds significantly depend on the alkyl chain length of the surfactants studied. The values of the diffusion coefficient of *N*-alkyl-*N*-methyl-*N*-(3-sulfopropyl)-6-(*N*-alkyl-*N*-methylamino)hexylammonium chloride tend to decrease as the surfactant concentration is increased.
